# LDH-stabilized ultrasmall iron oxide nanoparticles as a platform for hyaluronidase-promoted MR imaging and chemotherapy of tumors

**DOI:** 10.7150/thno.42906

**Published:** 2020-02-03

**Authors:** Ni Zhang, Yue Wang, Changchang Zhang, Yu Fan, Du Li, Xueyan Cao, Jindong Xia, Xiangyang Shi, Rui Guo

**Affiliations:** 1 State Key Laboratory for Modification of Chemical Fiber and Polymer Materials, International Joint Laboratory for Advanced Fiber and Low-dimension Materials, College of Chemistry, Chemical Engineering and Biotechnology, Donghua University, Shanghai 201600, People's Republic of China; 2 Department of Radiology, Shanghai Songjiang District Central Hospital, Shanghai 201600, People's Republic of China

**Keywords:** layered double hydroxide, ultra-small iron oxide, hyaluronic acid, hyaluronidase, theranostic nanoplatform

## Abstract

Development of unique theranostic nanoplatforms for tumor imaging and therapy remains an active topic in current nanomedicine. Here, we designed a novel targeted theranostic nanoplatform for enhanced *T_1_*-weighted magnetic resonance (MR) imaging-guided chemotherapy by constructing layered double hydroxide (LDH)-stabilized ultrasmall iron oxide (Fe_3_O_4_) nanoparticles with hyaluronic acid (HA) modified as targeting agents, and anticancer drug doxorubicin (DOX) loaded with a high loading efficiency.

**Methods**: The structure and release property of LDH-Fe_3_O_4_-HA/DOX nanoplatforms were characterized systematically. B16 melanoma cells with CD44 receptors overexpressed were used as model cells to determine the biocompatibility, targeting capability, and therapeutic efficiency of nanoplatforms. For *in vivo* experiment, hyaluronidase (HAase) pretreatment was combined with nanoplatform administration to investigate the MR imaging and chemotherapeutic effect.

**Results**: The LDH-Fe_3_O_4_-HA nanohybrids possess good colloidal stability and cytocompatibility, display an *r_1_* relaxivity 10-fold higher than the pristine ultrasmall Fe_3_O_4_ (4.38 mM^-1^ s^-1^ *vs* 0.42 mM^-1^ s^-1^), and could release drug in a pH-responsive manner. *In vitro* experiments demonstrate that LDH-Fe_3_O_4_-HA/DOX nanohybrids are able to specifically target B16 cells overexpressing CD44 receptors and effectively release DOX to nucleus. *In vivo* results show that with the pretreatment of tumor tissue by HAase to degrade the overexpressed HA in extra-cellular matrix, the designed nanoplatforms have a better tumor penetration for significantly enhanced MR imaging of tumors and tumor chemotherapy with low side effects.

**Conclusion**: The designed LDH-Fe_3_O_4_-HA/DOX nanohybrids may be developed as a novel targeted theranostic nanoplatform for enhanced *T_1_*-weighted MR imaging-guided chemotherapy of CD44 receptor-overexpressing tumors.

## Introduction

Development of theranostic nanoplatforms for enhanced tumor imaging and targeted chemotherapy has been a hot spot of cancer treatment in current decades, since the combination of diagnosis and anticancer drug within one single nanoplatform could observe the biodistribution of nanomedicines and monitor the therapeutic effect in real-time 1-4. Among different imaging methods, magnetic resonance (MR) imaging is a popular and effective noninvasive imaging tool in clinical diagnosis of tumors due to its precise soft tissue contrast, and especially with the high-performance contrast agents, its sensitivity and reliability could be significantly improved. Ultrasmall iron oxide nanoparticles (NPs) have attracted tremendous attention as a *T_1_*-weighted MR contrast agent due to their high longitudinal relaxivity (*r_1_*) and good biocompatibility in comparison with commercialized Gadolinium-based contrast agents 5-8. Various polymers were conjugated on the surface of ultrasmall Fe_3_O_4_ NPs to improve their stability under biological condition, and targeting moiety were further modified to facilitate the specific uptake of NPs by cancer cells for the early detection of tumors 9-11. More recently, graphene oxide (GO) were used as templates to grow ultra-small Fe_3_O_4_ NPs in situ and load anticancer drug doxorubicin (DOX) with pH-responsive release property for theranostic functions 12. However, it is still an open challenge to construct targeted theranostic nanoplatforms with good colloidal stability and biocompatibility, a high *r_1_* relaxivity for accurate diagnosis and specific tumor accumulation *in vivo* for effective chemotherapy 13, 14.

Layered double hydroxide (LDH), as a biocompatible and biodegradable 2D nanomaterial, is ideal to construct such multifunctional nanoplatform 15. By the virtue of the unique hydroxide layered structure, LDH possess a high specific surface area and anion exchange capacity for drug loading, and could be biodegraded under mildly acidic circumstance to facilitate drug release 16. Moreover, LDH could be used to stabilize some functional nanoparticles, such as Au NPs or iron oxide NPs 17, 18. For instance, Bahadur et al. decorated Au NPs on the surface of LDH, and the formed LDH-Au exhibited good biocompatibility and colloidal stability for photothermal therapy 19. Gu et al. demonstrated that the *r_2_* relaxivity of iron oxide NPs increased after stabilized by LDH, probably due to that the OH groups of LDH structure could lead to an increased number of water molecules around iron oxide NPs for enhancing the *T_2_*-weighted MR effect 20. In addition, LDH could be easily coated with phosphonic acid terminated PEG or natural biomolecules such as serum albumin to increase their stability in body, and could also conjugate with targeting agents to increase the accumulation of NPs at tumor sites 21. Choy et al. proved that folic acid modified LDH could deliver more amount of therapeutic agents to tumor and exhibit 3 folds higher suppression of tumor volume than unmodified LDH 22. Therefore, LDH would be an excellent carrier candidate to load drugs and ultra-small iron oxide NPs to build a theranostic nanoplatform for targeted MR imaging and chemotherapy.

Hyaluronic acid (HA) is a kind of natural and linear polysaccharide, and has been extensively utilized as a targeting moiety for cancer cells overexpressing CD44 receptors in tumor imaging 23-25, gene delivery 26, and drug delivery applications 27, 28. Due to its outstanding biocompatibility and specific targeting ability, HA modification could effectively improve the stability, prolong the circulation time and enhance the uptake of NPs by tumor cells via CD44 receptor-mediated endocytosis 29, 30. Moreover, HA is also a key component of condensed tumor extracellular matrix (ECM). The overabundant HA in tumor tissues is the major biological barrier to hinder the penetration of medicines, resulting in the low therapeutic efficacy of chemotherapy and a poor survival rate 31. To overcome this, hyaluronidase (HAase) was introduced to treat tumors for temporarily reducing the viscosity of the intercellular substance and enhancing the penetration and diffusion of nanomedicines in tumor tissue 32, 33. Liu et al. illustrated that the pretreatment of HAase could lower the interstitial flow pressure and enhance the perfusion inside the tumor, which may increase the tumor uptake of NPs and improve the efficacy of *in vivo* photodynamic therapy 33. Chen et al. demonstrated that PD-L1 gene silence could be more efficiently achieved by the combination of HAase due to the increase of the penetration of NPs by the efficient degradation of HA in tumors 32. Therefore, in this study, HAase will be utilized to pretreat the tumors to promote the penetration of tumor tissue, and then the specific uptake of HA modified nanoplatforms would be increased for targeted theranostics.

**Scheme 1:**
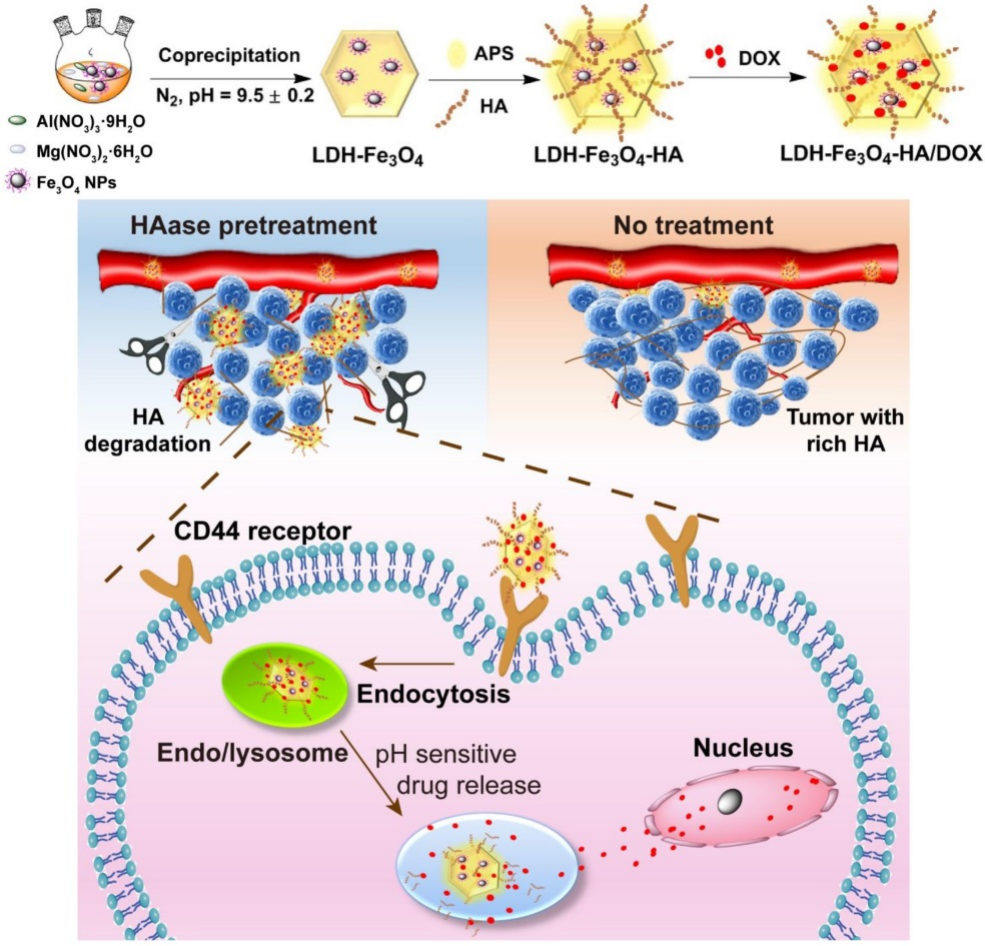
Synthetic procedure and theranostic mechanism of LDH-Fe_3_O_4_-HA/DOX nanoplatforms.

Herein, we developed a novel theranostic nanoplatform for *T_1_*-weighted MR imaging and chemotherapy against tumors overexpressing CD44 receptors. In this system, ultra-small Fe_3_O_4_ nanoparticles were stabilized on LDH nanosheets with an enhanced *r_1_* relaxivity, and HA was modified on the surface of LDH-Fe_3_O_4_ as targeting moiety, followed by the loading of anticancer drug DOX (Scheme 1). To our knowledge, this is the first report to the combination of HAase pretreatment with HA-modified LDH nanoparticles for targeted* T_1_*-weighted MR imaging and chemotherapy.

## Materials and Methods

### Materials

All materials and cells used in the experiment were offered by commercial companies as our previous report (details can be found in Supporting Information).

### Preparation of ultra-small Fe_3_O_4_ NPs

Ultrasmall iron oxide nanoparticles were synthesized according to our previous work by a solvothermal route method 34. Firstly, FeCl_3_ (648 mg) was dissolved into 40 mL of diethylene glycol (DEG). Na_3_Cit · 2H_2_O (471 mg) was added into the mixture solution under stirring at 80 °C for 2 h. Then, cooling down mixture solution to room temperature, anhydrous sodium acetate (1312 mg) was dissolved into above solution. Finally, the mixture was hydrothermally treated at 200 °C for 4 h. After cooling down, the black oily liquid was collected by centrifugation (7512 g, 15 min) and washed thoroughly with anhydrous ethanol. Fe_3_O_4_ nanoparticles were obtained by vacuum drying for further use.

### Synthesis of LDH-Fe_3_O_4_ NPs

Fe_3_O_4_ NPs (20 mg) were dispersed in 10 mL deionized water. Then, Fe_3_O_4_ solution was mixed with Mg(NO_3_)_2_·6H_2_O (1155 mg) and Al(NO_3_)_3_·9H_2_O (845 mg), and the final pH of mixture was adjusted to 9.5 ± 0.2 by adding NaOH (1.0 M) solution and further aged for 48 h. The synthesized LDH-Fe_3_O_4_ nanoparticles were separated by centrifugation (4393 g, 5 min), and washed with deionized water thoroughly before lyophilization.

### Synthesis of LDH-Fe_3_O_4_-HA NPs

3-aminopropyl triethoxysilane (APS, 0.1 mL) was dropped into LDH-Fe_3_O_4_ solution (20 mL, 5 mg/mL) and stirred vigorously under nitrogen at room temperature for 12 h. Then LDH-Fe_3_O_4_-APS NPs was purified by centrifugation to remove unconjugated APS, and dispersed in deionized water. The carboxyl group of HA (10 mL, 10 mg/mL) was activated by 1-ethyl-3-(3-dimethyl aminopropyl)-carbodiimide (EDC, 1 mL, 15 mg/mL) and N-hydroxysuccinimide (NHS, 1 mL, 10 mg/mL) at room temperature for 3 h. Then, the activated HA was mixed with LDH-Fe_3_O_4_-APS and reacted for 2 days. Finally, LDH-Fe_3_O_4_-HA was purified by centrifugation to remove excess HA.

### Drug loading and releasing

LDH-Fe_3_O_4_-HA (5 mL, 2 mg/mL) was blended with DOX·HCl solution (10 mL, 2 mg/mL) under darkness for 48 h at room temperature. Then the solution was purified by centrifugation (7512 g, 15 min), and the precipitate was washed 3 times with water to obtain drug-loaded LDH-Fe_3_O_4_-HA/DOX.

The structure and morphology of LDH-Fe_3_O_4_-HA/DOX were characterized by different techniques, and the drug loading and release properties were also studied (Details in Supporting Information).

### *In vitro* and *in vivo* assays

*In vitro* cytotoxicity, cellular uptake, *in vivo* MR imaging and therapeutic effect were also evaluated (details of methods can be found in Supporting Information).

## Results and Discussion

### Synthesis and characterization of LDH-Fe_3_O_4_-HA/DOX

**Figure 1:**
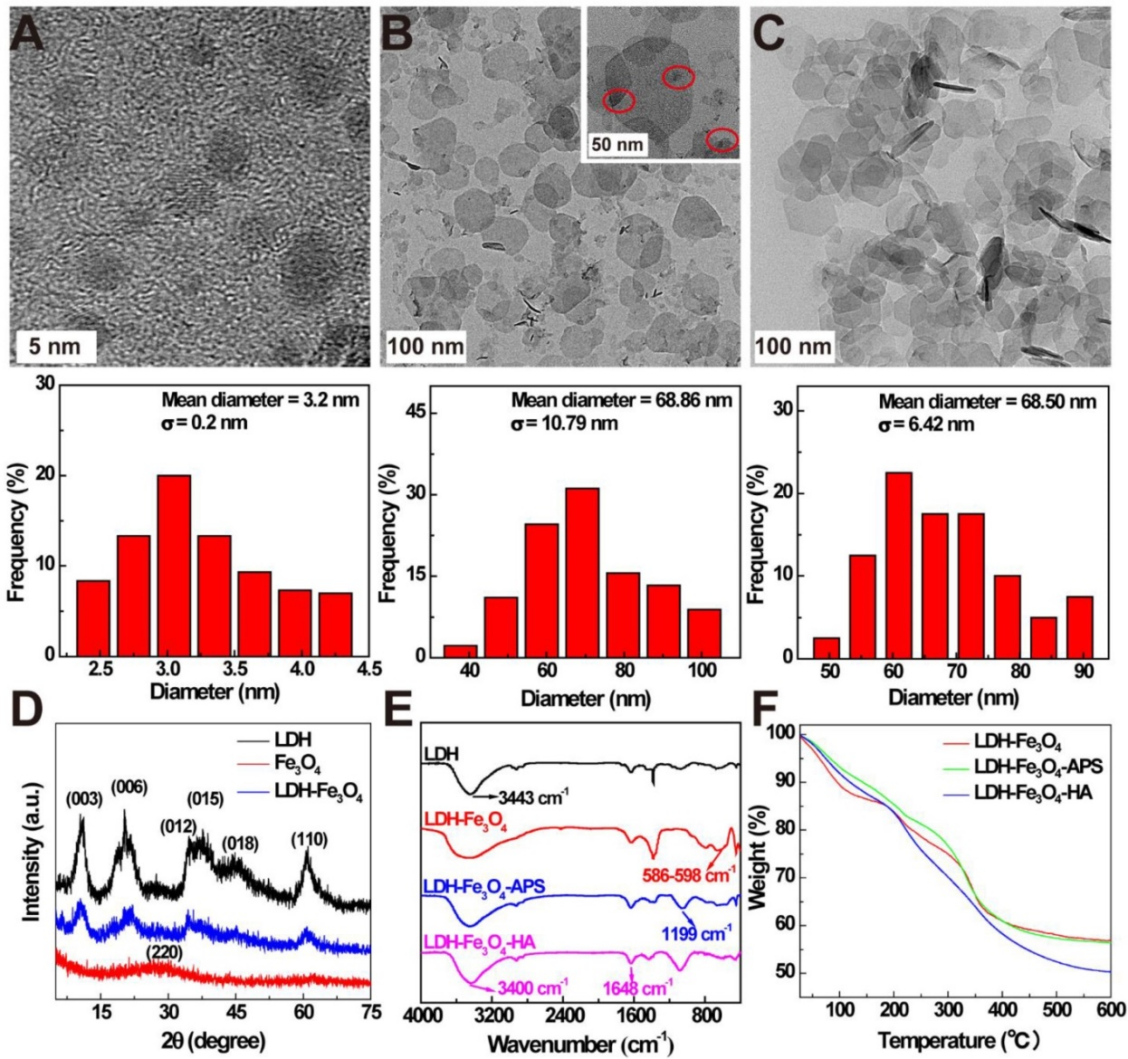
TEM images and size distribution histograms of (A) ultrasmall Fe_3_O_4_, (B) LDH-Fe_3_O_4_ and (C) LDH. (D) XRD patterns of LDH, Fe_3_O_4_, and LDH-Fe_3_O_4_. (E) FT-IR spectra and (F) TGA curves of LDH-Fe_3_O_4_, LDH-Fe_3_O_4_-APS, and LDH-Fe_3_O_4_-HA.

In this study, ultra-small Fe_3_O_4_ NPs with a mean diameter of 3.2 ± 0.2 nm were firstly prepared by a solvothermal route according to our previous work (Figure 1A) 34. Then, LDH were synthesized via the coprecipitation method to stabilize Fe_3_O_4_ NPs, and the formed LDH-Fe_3_O_4_ NPs have a relatively uniform disk shape with diameter of 68.8 ± 10.7 nm (Figure 1B), which is similar to that of LDH (68.5 ± 6.4 nm, Figure 1C, SEM result in Figure S1). Obviously, Fe_3_O_4_ NPs with high density were present as black dots on the grey LDH nanosheets. Moreover, X-ray diffraction (XRD) was applied to characterize the patterns of LDH and LDH-Fe_3_O_4_. As shown in Figure 1D, both LDH and LDH-Fe_3_O_4_ are well-crystalized with reflections of (003), (006), (009), (015), (018) and (110) planes, and their (003) basal spacing was calculated to be 0.78 nm (Table S1), which is similar to the LDH synthesized in literature 22. This result demonstrated that the loading of ultra-small Fe_3_O_4_ NPs did not change the inherent structure of LDH, which may endow a huge potential in drug encapsulation and controlled drug release.

Then silane coupling agents were modified on the surface of LDH-Fe_3_O_4_ nanoparticles to introduce active amino groups, followed by the conjugate of HA as targeting agents for tumor cells overexpressing CD44 receptors. The stepwise synthesis was confirmed by FT-IR results in Figure 1E. Compared with pristine LDH, LDH-Fe_3_O_4_ exhibited a strong band at 586-598 cm^-1^, which is assigned to the Fe-O vibration of loaded Fe_3_O_4_ NPs 34. In the spectra of LDH-Fe_3_O_4_-APS, the emerging peak at 1199 cm^-1^ illustrated the successful introduction of -NH_2_ onto the LDH by silane coupling agents 22. For LDH-Fe_3_O_4_-HA, the broad stretching vibration located at 1648 cm^-1^ (-CO-NH-) could be ascribed to the covalent linkage of HA with LDH-Fe_3_O_4_-APS, and the increased absorption at 3400 cm^-1^ may be assigned to -NH and -OH stretching vibrations 35. Moreover, TGA results further proved the successful synthesis of LDH-Fe_3_O_4_-HA (Figure 1F). Compared with LDH-Fe_3_O_4_, LDH-Fe_3_O_4_-APS NPs had a slightly additional weight loss of 1.89% from 200-600 °C due to the thermal decomposition of APS on surface. After HA modification, the weight loss of LDH-Fe_3_O_4_-HA is calculated to be 13.28%, which could be attributed to the conjugation of HA. Therefore, LDH-Fe_3_O_4_-HA was successfully synthesized as design.

The hydrodynamic diameters and surface potentials of nanoparticles were measured by dynamic light scattering (DLS) (Table 1 and Figure S2). Pristine LDH displayed a mean hydrodynamic diameter of 107.2 nm and a high positive zeta potential of 36.60 mV. After the loading of negative charged Fe_3_O_4_ NPs by electronic interactions (-30.20 mV), the zeta potential of LDH-Fe_3_O_4_ reduced to 26.54 mV. Finally, the surface potential turned to -13.30 mV after the conjugation of HA, indicating that LDH-Fe_3_O_4_-HA were synthesized successfully. LDH-Fe_3_O_4_-HA NPs possess a hydrodynamic diameter of 325.1 nm due to the HA chain on surface, and could keep stable in water, saline, and RPMI 1640 medium (containing 10% FBS) for 1 month, indicating their good colloidal stability for potential biomedical applications (Figure S3).

**Figure 2:**
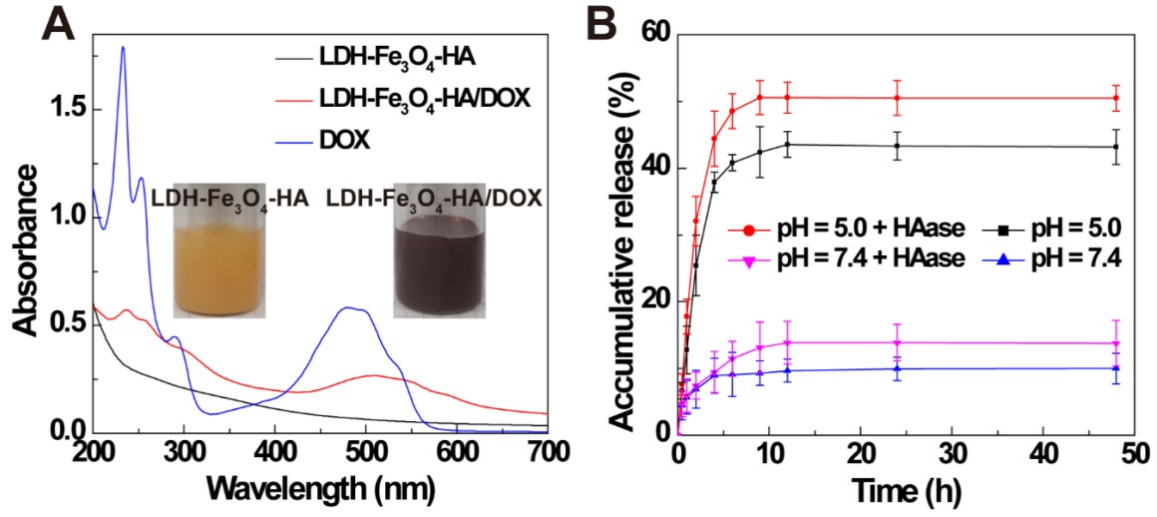
(A) UV-vis spectra of LDH-Fe_3_O_4_-HA, LDH-Fe_3_O_4_-HA/DOX, and DOX. (B) Accumulative release of DOX from LDH-Fe_3_O_4_-HA/DOX in buffer solution (pH = 7.4 and pH = 5.0 at 37 °C) in the presence or absence of HAase (1 mg/mL).

Finally, anti-tumor drug DOX was loaded on LDH-Fe_3_O_4_-HA by physical mixture. The formed LDH-Fe_3_O_4_-HA/DOX displayed a typical red color and an obvious absorption at 480 nm in UV-vis spectra (Figure 2A), indicating the successful loading of DOX on LDH-Fe_3_O_4_-HA. The drug loading efficiency of LDH-Fe_3_O_4_-HA was calculated to be as high as 97.81% and loading capacity as 57.65%. Importantly, LDH-Fe_3_O_4_-HA/DOX exhibited well dispersed colloidal stability with 345.5 nm in mean hydrodynamic diameter and -9.10 mV in zeta potential.

The drug release property of LDH-Fe_3_O_4_-HA/DOX was investigated under pH 5.0 and 7.4 to simulate the physiological environment and tumor site. As shown in Figure 2B, 42.88% of DOX was released from nanocomplexes at pH = 5.0 within 9 h, while only 9.15% drug was released at pH 7.4. The pH-sensitive release property may reduce the release of drug during body circulation and lower the side effect of nanocomplexes* in vivo*. Moreover, the influence of HAase on drug release was also investigated, since HAase overexpressed in the endo-lysosome may facilitate the release of drug by the degradation of HA 36-39. It is obvious that over 50% of drug was released at pH 5.0 within 9 h, suggesting that the drug release may be greatly accelerated with HAase in endo-lysosome after uptake by cancer cells. Finally, the surface potential of LDH-Fe_3_O_4_-HA after HAase treatment was monitored to investigate the degrade of HA on NPs at different pH circumstances (Figure S4). The zeta potential of LDH-Fe_3_O_4_-HA NPs kept negative charged after 4 h under pH 7.4, but turned sharply to positive charged within 15 min under pH 5.0, indicating that HA on LDH-Fe_3_O_4_-HA NPs may be quickly degraded by HAase under weak acid condition 40, 41. Hence, HA as the outer corona of LDH-Fe_3_O_4_-HA could increase the longevity during blood circulation, enhance their accumulation by CD44 receptor recognition, and be detached at HAase-rich cellular endo-lysosome for quick drug release. In sum, LDH-Fe_3_O_4_-HA/DOX nanocomplexes could efficiently release drug at tumor by a pH-responsiveness and enzymatic hydrolysis behavior, thereby achieving better chemotherapeutic effect.

### *T_1_*-weighted MR imaging performance of LDH-Fe_3_O_4_-HA solution

**Figure 3:**
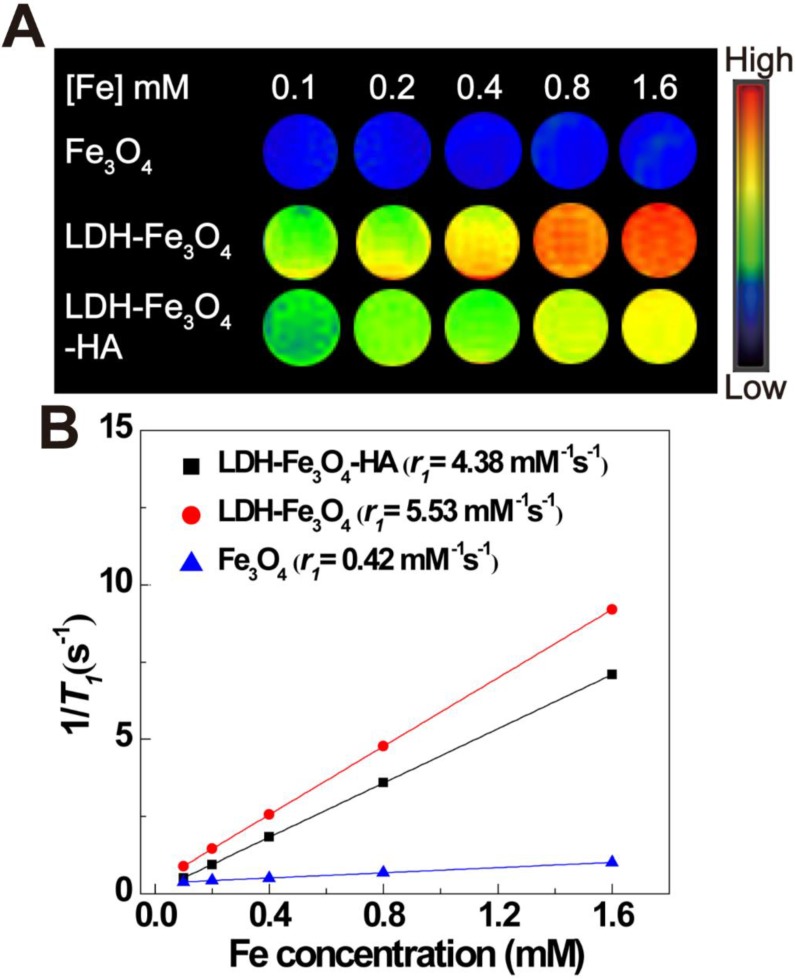
*T_1_*-weighted MR image (A) and linear fitting of 1/*T_1_* (B) in terms of Fe concentration of Fe_3_O_4_, LDH-Fe_3_O_4_ and LDH-Fe_3_O_4_-HA. The color bar from blue to red indicates the gradual increase in the MR signal intensity.

To explore the MR imaging capability of LDH-Fe_3_O_4_-HA, we implemented MR imaging experiments using Fe_3_O_4_, LDH-Fe_3_O_4_ and LDH-Fe_3_O_4_-HA solutions at various Fe concentrations. As shown in Figure 3A, the* T_1_*-weighted MR imaging intensities of all materials enhanced gradually with the increase of Fe concentration, indicating their potential as MR contrast agents. Importantly, the MR signal of LDH-Fe_3_O_4_ was much higher than that of Fe_3_O_4_ NPs at the same Fe concentration. By plotting the relaxation rate (*1/T_1_*) as a term of Fe concentration (Figure 3B), the *r_1_* relaxivity of LDH-Fe_3_O_4_ is calculated to be 5.53 mM^-1^ s^-1^, which was 13.2-fold higher than that of Fe_3_O_4_ (0.42 mM^-1^ s^-1^). The significant enhancement in *r_1_* value is owing to the existence of LDH, which may increase the spacing between Fe_3_O_4_ NPs and reduce the magnetic coupling between them, resulting in a smaller effective magnetic size than pure Fe_3_O_4_ NPs 20, 42. Since the low *r_2_*/*r_1_* ratio is a key parameter to confirm the strong *T_1_* contrast efficiency of MRI contrast agent 43, the *r_2_* value of LDH-Fe_3_O_4_-HA was also measured in Figure S5. The *r_2_*/*r_1_* ratio of LDH-Fe_3_O_4_-HA was calculated to be 0.94, which is relatively lower than other Fe_3_O_4_ based *T_1_* contrast agents reported previously 9. After the modification of HA and loading of DOX, the *r_1_* value of LDH-Fe_3_O_4_-HA and LDH-Fe_3_O_4_-HA/DOX remained as high as 4.38 mM^-1^ s^-1^ and 4.16 mM^-1^ s^-1^ (Figure S6), respectively. Hence, LDH-Fe_3_O_4_-HA have the potential to be a high-performance *T_1_*-weighted MR contrast agent.

### Cytotoxicity and Cellular uptake

**Figure 4:**
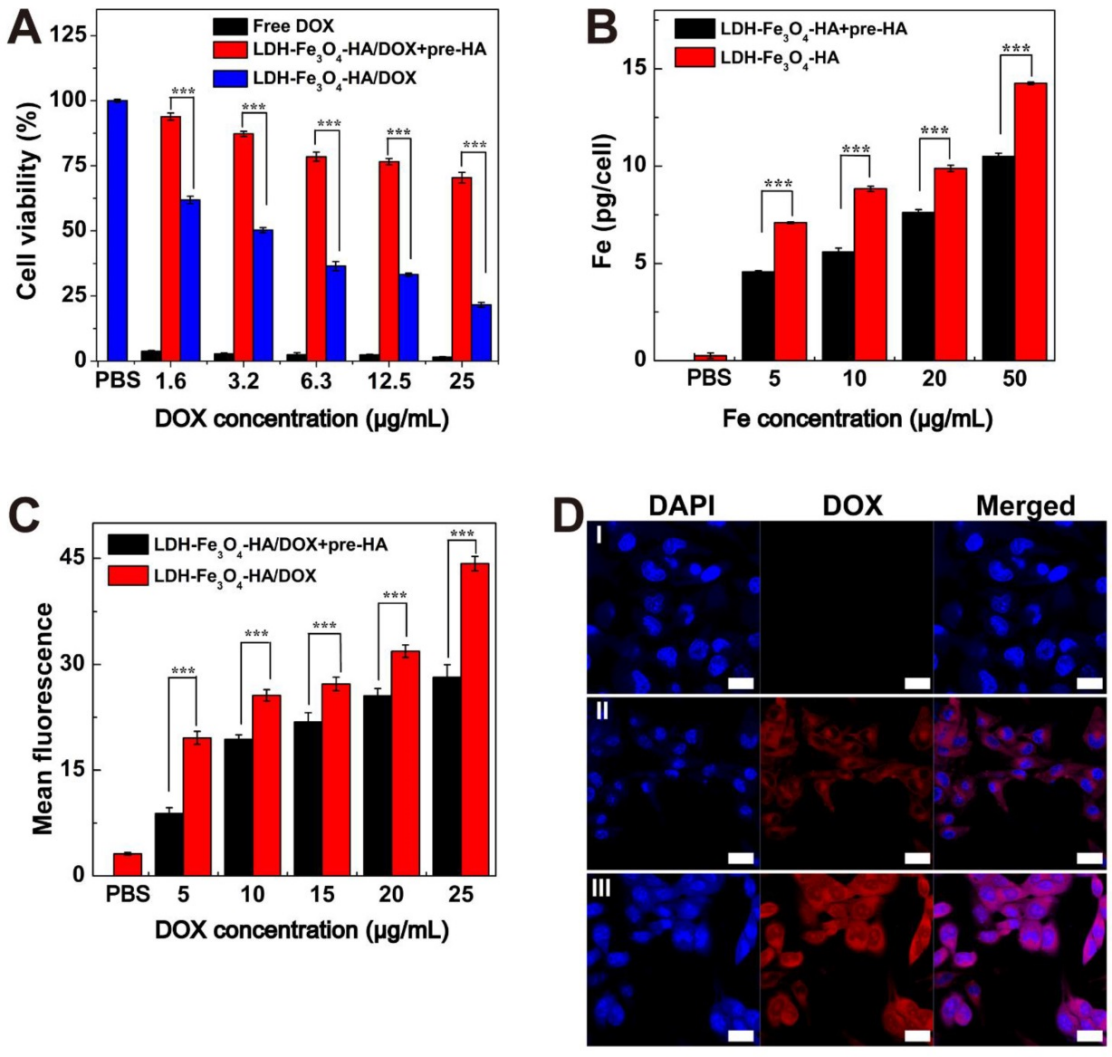
(A) Cell viability of B16 cells treated with free DOX and LDH-Fe_3_O_4_-HA/DOX at different concentrations of DOX for 48 h. (B) The cellular Fe concentration of B16 cells after treated with LDH-Fe_3_O_4_-HA at various Fe concentrations for 4 h. (C) Flow cytometry of B16 cells treated with LDH-Fe_3_O_4_-HA/DOX at different DOX concentrations for 4 h. (D) Confocal images of B16 cells treated with PBS (I), LDH-Fe_3_O_4_-HA/DOX+pre-HA (II), and LDH-Fe_3_O_4_-HA/DOX (III) (c_DOX_ = 5 μg/mL) for 4 h (Scale bar: 20 μm).

B16 melanoma cells with CD44 receptors over-expressed were chosen as model cells in this study. To verify the specific interaction of LDH-Fe_3_O_4_-HA to CD44 receptors, competitive binding experiments were carried out by pre-incubating B16 cells with free HA (2.0 mM) for 2 h before NPs treatment, denoted as pre-HA group 44-46. Firstly, the biocompatibility of LDH-Fe_3_O_4_-HA was investigated by CCK-8 assay (Figure S7A). It is clear that 94% of B16 cells remained alive after incubation with LDH-Fe_3_O_4_-HA for 24 h, indicating their excellent biocompatibility in a given concentration range. Then the antitumor activity of drug-loaded nanocomplexes was investigated by measuring the cell viability after incubation for 48 h (Figure 4A). Both free DOX and LDH-Fe_3_O_4_-HA/DOX group displayed a dose-dependent cytotoxicity in treating B16 cells. Free DOX displayed a better inhibition capability than LDH-Fe_3_O_4_-HA/DOX, which could be attributed to the relatively higher effective drug concentration in free DOX group than nanocomplexes with slow drug release rate and limited actual amount of drug to exert tumor inhibition effect. More importantly, the pre-HA group showed a negligible inhibition effect, which may be due to the limit cell uptake of NPs by the blocking effect of excessive free HA. It is worth mentioning that with the prolongation of incubation time from 24 h to 48 h, the cell viability of B16 cells treated with nanocomplexes decreased dramatically due to the sustained release of DOX (p < 0.001, Figure S7B). Considering the pH-sensitive and HAase-accelerated release property, the synthesized LDH-Fe_3_O_4_-HA/DOX could exhibit long-term therapeutic effect and much lower side effects than free DOX.

In addition, the targeting uptake of LDH-Fe_3_O_4_-HA was evaluated by measuring the Fe concentration in B16 cells after co-incubation for 4 h by ICP-OES. As shown in Figure 4B, the Fe concentration in B16 cells exhibited concentration-dependent behavior, indicating higher LDH-Fe_3_O_4_-HA NPs uptake rate by B16 cells. More importantly, the Fe concentration in pre-HA group was significantly lower than that of LDH-Fe_3_O_4_-HA group (p < 0.001) at the same NPs concentration, verifying that the cell uptake of NPs could be inhibited by the blocking of specific interaction between LDH-Fe_3_O_4_-HA/DOX NPs and CD44 receptors on cell surface by the excessive free HA. This result demonstrated that LDH-Fe_3_O_4_-HA could act as targeted nanocarriers and accumulate in CD44 receptors overexpressed cancer cells *via* receptor-mediated endocytosis.

Finally, the targeted drug delivery of LDH-Fe_3_O_4_-HA/DOX was evaluated by flow cytometry (Figure 4C and Figure S8) and confocal microscopy (Figure 4D). With the increase of DOX concentration, both LDH-Fe_3_O_4_-HA/DOX and pre-HA group presented an enhanced fluorescence intensity, but LDH-Fe_3_O_4_-HA/DOX showed a significant higher fluorescence intensity and apparent fluorescent signal shift in comparison with pre-HA group (p < 0.001), indicating the targeting specificity of LDH-Fe_3_O_4_-HA/DOX towards B16 cells overexpressing CD44 receptors. Moreover, cellular uptake and drug release rate could be directly monitored via fluorescent signals of DOX in confocal images. When incubated with the same concentration of LDH-Fe_3_O_4_-HA/DOX, free HA pretreatment may obviously decrease the red fluorescence in B16 cells both in cytoplasm and nucleus, indicating the weakening of CD44-mediated endocytosis of LDH-Fe_3_O_4_-HA/DOX by the competition of free HA 47, 48. This phenomenon validated that LDH-Fe_3_O_4_-HA/DOX can actively target B16 cells via CD44-mediated endocytosis and DOX can be effectively released for treatment. In sum, LDH-Fe_3_O_4_-HA/DOX could be a biocompatible and targeted theranostic nanoplatform for *T_1_*-weighted MR imaging and chemotherapy of cancer cells overexpressing CD44 receptors.

### *In vivo* MR imaging and biodistribution of LDH-Fe_3_O_4_-HA NPs

**Figure 5:**
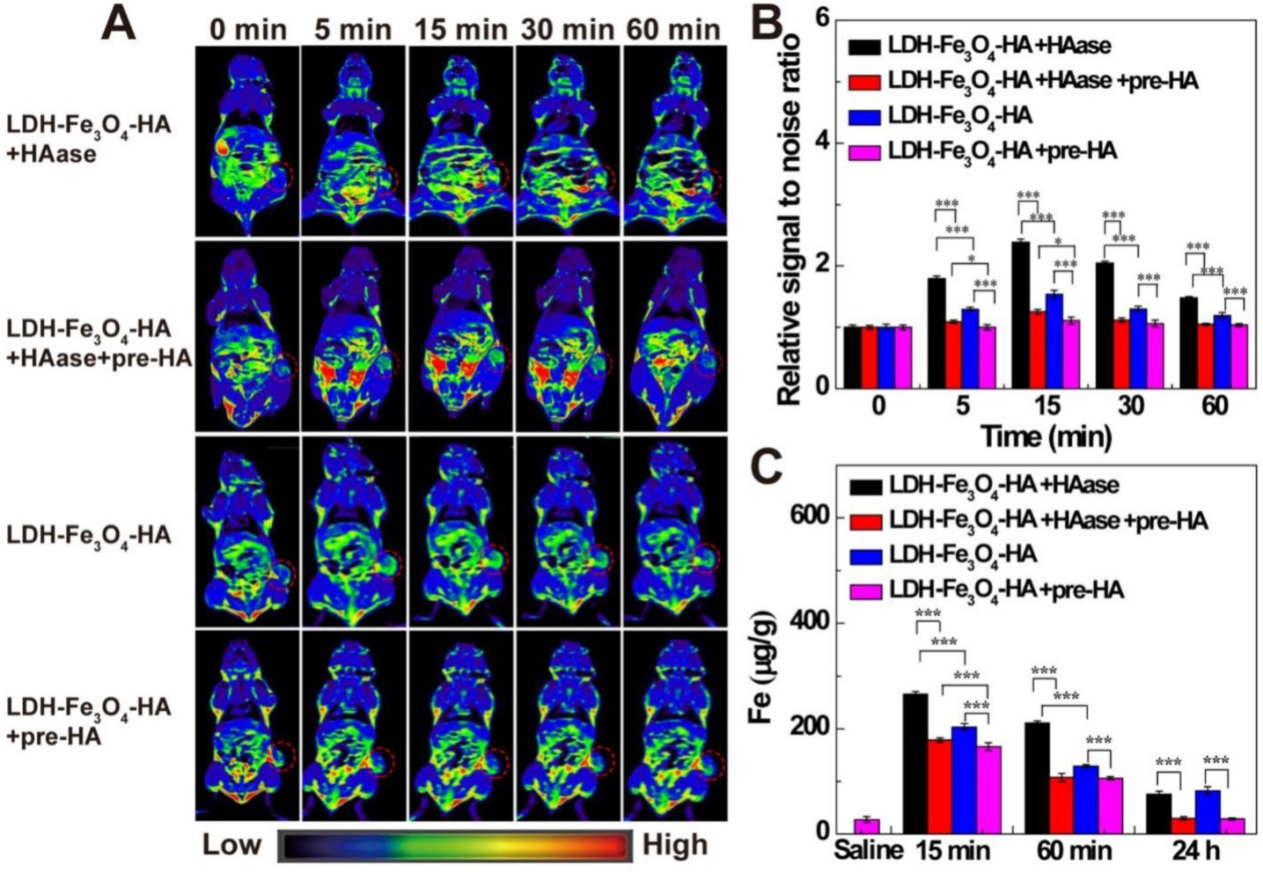
(A) *In vivo T_1_*-weighted MR images; (B) the relative MR signal intensity and (C) the Fe concentration in tumors at different time points after administration of LDH-Fe_3_O_4_-HA+HAase, LDH-Fe_3_O_4_-HA+HAase+pre-HA, LDH-Fe_3_O_4_-HA+pre-HA and LDH-Fe_3_O_4_-HA ([Fe] = 500 μg/mL, 0.2 mL saline for each mouse).

To investigate the targeted MR imaging performance *in vivo*, B16 melanoma tumor bearing C57BL/6 mice were established and divided into LDH-Fe_3_O_4_-HA group, LDH-Fe_3_O_4_-HA+pre-HA group, LDH-Fe_3_O_4_-HA+HAase and LDH-Fe_3_O_4_-HA+HAase+pre-HA group. For pre-HA and HAase group, HA (24 mg, 100 μL saline) and HAase (0.1 mg, 50 μL saline) were intratumorally injected 1 h before the intravenous injection of LDH-Fe_3_O_4_-HA, respectively. To evaluate the targeted property of LDH-Fe_3_O_4_-HA after HAase pretreatment, free HA was injected 1 h after the administration of HAase at tumor, and then LDH-Fe_3_O_4_-HA nanoparticles were injected intravenously. The *in vivo* MR images of different groups were shown in Figure 5A. Both LDH-Fe_3_O_4_-HA+HAase group and LDH-Fe_3_O_4_-HA group exhibited a remarkable contrast enhancement at tumor after administration, while there was a relatively weak increase of MR signal intensity at tumor of LDH-Fe_3_O_4_-HA+pre-HA and LDH-Fe_3_O_4_-HA+HAase+pre-HA group. This result further verified that the modified HA could facilitate the specific accumulation and efficient uptake of NPs in tumor *via* the CD44 receptor-mediated endocytosis. Impressively, LDH-Fe_3_O_4_-HA+HAase group displayed a much higher contrast and long-term MR image of tumor than other groups. Then, the relative signal-to-noise ratio (SNR) values were measured to quantitatively analyze MR signal enhancement (Figure 5B). All groups displayed the highest MR signal enhancement at 15 min after injection. It is interesting to find that HA pretreatment could diminish the MR signal at tumor significantly (p < 0.001), which may be attributed to the block of the specific interaction between HA on NPs with CD44 receptors on B16 cell surface, while HAase pretreatment could increase the MR signal at tumor significantly, probably due to the increased penetration by breakdown the excess HA in ECM structure 32, 33. As a result, the MR signal of LDH-Fe_3_O_4_-HA+HAase group at tumor was 1.56 folds higher than that of LDH-Fe_3_O_4_-HA, and about 2 folds higher than those of LDH-Fe_3_O_4_-HA+pre-HA and LDH-Fe_3_O_4_-HA+HAase+pre-HA group. And even after 60 min, LDH-Fe_3_O_4_-HA+HAase group could still maintain their excellent imaging performance over other groups (p < 0.001), demonstrating the combination of HAase pretreatment and HA modification could enhance the penetration and specific accumulation of LDH-Fe_3_O_4_-HA NPs in the center of tumor. Hence, with the aid of HAase pretreatment, the developed LDH-Fe_3_O_4_-HA NPs could accumulate at tumor site efficiently and target CD44 overexpressed tumor cells specifically for enhanced *T_1_*-MR imaging and delivery of therapeutic agents as well.

Moreover, the biodistribution and metabolism of LDH-Fe_3_O_4_-HA NPs were evaluated with ICP-OES by analyzing Fe contents in tumor, heart, liver, spleen, lungs, and kidneys at different time intervals (Figure 5C and Figure S9). In consistent with MR imaging result, the Fe concentration at tumor reached the maximum at 15 min postinjection, and LDH-Fe_3_O_4_-HA+HAase group exhibited the highest Fe concentration among all groups (p < 0.001). HAase pretreatment could significantly enhance the Fe concentration at tumor, indicating the increasing accumulation of LDH-Fe_3_O_4_-HA NPs at tumor site by the hydrolysis of excess HA in ECM. And the *in vivo* targeting property of LDH-Fe_3_O_4_-HA could be demonstrated by the significantly lower Fe concentration at tumor of HA pretreatment group in comparison with NP groups (p < 0.001). After 24 h, the Fe concentration in all major organs decreased to a normal level, indicating that the injected nanoparticles were almost completely cleared up from the body. In contrast, the Fe concentration in tumors of LDH-Fe_3_O_4_-HA group and LDH-Fe_3_O_4_-HA+HAase group is still significantly higher than that of pre-HA group and HAase+pre-HA group. This verified that LDH-Fe_3_O_4_-HA could specifically accumulate and retain at tumor site for a long period, which could be benefit for the sustained chemotherapy of theranostic nanoplatform. Therefore, LDH-Fe_3_O_4_-HA NPs could be specifically accumulated at tumor for a relatively long time and finally metabolized from the body.

### *In vivo* antitumor activity of LDH-Fe_3_O_4_-HA/DOX

**Figure 6:**
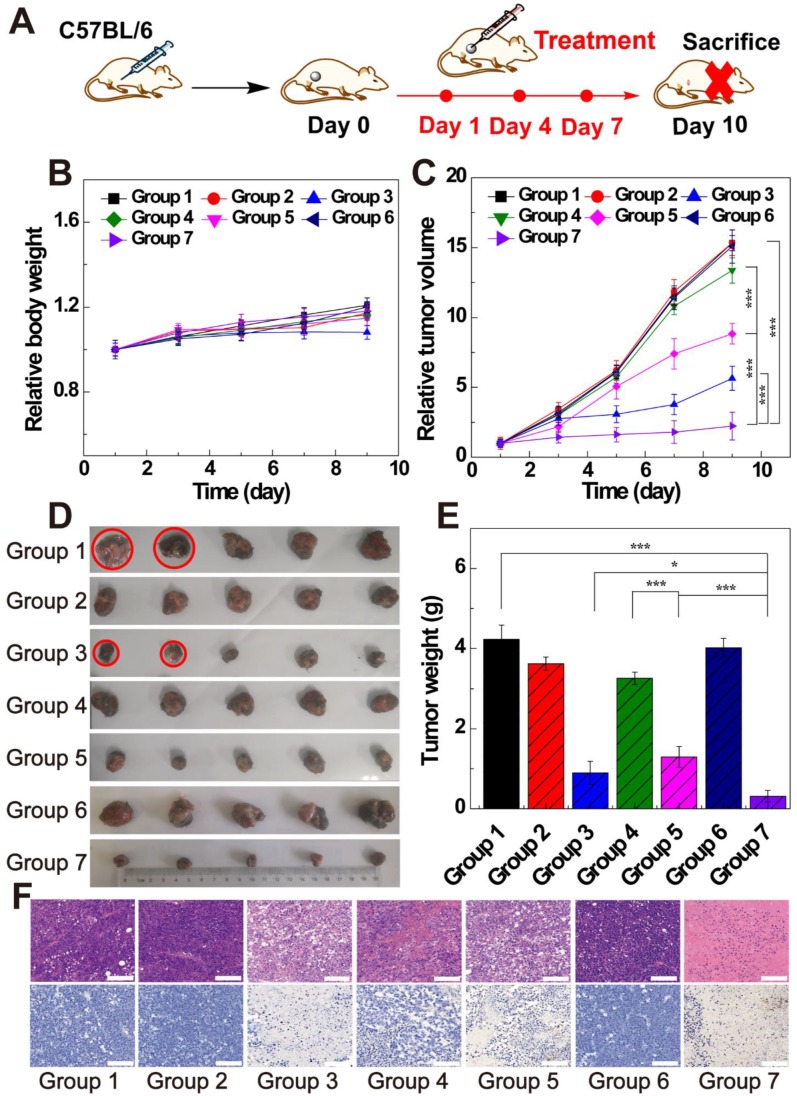
(A) The therapeutic schedule of *in vivo* experiment; (B) the relative body weight and (C) the relative tumor volume of mice after treated by different groups (n = 5). (D) Representative photographs of tumor tissues and (E) the average tumor weight of different groups. (F) H&E staining and TUNEL staining of tumor sections of different groups (scale bar: 100 μm). (Group 1, saline; Group 2, LDH-Fe_3_O_4_-HA; Group 3, free DOX; Group 4, LDH-Fe_3_O_4_-HA/DOX+pre-HA; Group 5, LDH-Fe_3_O_4_-HA/DOX; Group 6, HAase; Group 7, LDH-Fe_3_O_4_-HA/DOX+ HAase.)

To verify the *in vivo* therapeutic effect of LDH-Fe_3_O_4_-HA/DOX, B16 melanoma tumor bearing C57BL/6 mice were randomly divided into seven groups when the tumor sizes reached about 200 mm^3^. Each group (n = 5) is scheduled to take intratumoral administration of different materials on Day 1, 4, and 7 (Figure 6A): Group 1, saline; Group 2, LDH-Fe_3_O_4_-HA; Group 3, free DOX; Group 4, LDH-Fe_3_O_4_-HA/DOX+pre-HA; Group 5, LDH-Fe_3_O_4_-HA/DOX; Group 6, HAase; Group 7, LDH-Fe_3_O_4_-HA/DOX+HAase. For Group 4 and 7, HA (24 mg, 100 μL saline) and HAase (0.1 mg, 50 μL saline) were intratumorally injected 1 h before the administration of NPs, respectively. The body weight and tumor volume of mice were measured every other day as shown in Figure 6B and 6C. Among all groups, only the body weight of mice in free DOX group displayed a declining trend during treatment, probably due to the severe side effect of free DOX. The tumor sizes of saline, HAase and LDH-Fe_3_O_4_-HA groups increased about 15-fold, suggesting that HAase and LDH-Fe_3_O_4_-HA had almost no therapeutic effect in tumor treatment. For drug-loaded groups, LDH-Fe_3_O_4_-HA/DOX+HAase group could significantly inhibit the relative tumor growth in comparison with LDH-Fe_3_O_4_-HA/DOX group and pre-HA treated group (2.23 *vs* 8.83 *vs* 13.36, p < 0.001). Their superior *in vivo* therapeutic effect should be attributed to the enhanced accumulation of nanoparticles in tumor and the efficient uptake *via* the specific interaction between HA and CD44 receptors overexpressed on cell surface. Although free DOX could inhibit tumor growth, two mice in free DOX group were dead on day 5 and day 7 during treatment possibly due to its severe side effect. Notably, LDH-Fe_3_O_4_-HA/DOX+HAase group could suppress the tumor growth more effectively than free DOX (2.23 *vs* 5.64, p < 0.001), which should be due to the enhanced penetration, specific accumulation and long retention of LDH-Fe_3_O_4_-HA/DOX in tumor, leading to sustained release of drug 47, 48. Moreover, no mice in nanoplatform groups died during treatment, indicating their good biocompatibility. At the end of treatment, all mice were sacrificed, and the tumors and organs were collected for further investigation. Figure 6D and 6E represented photographs and average weight of tumors in each group. It is worth mentioning that, two mice in saline group were dead due to the aggressive growth of tumor (red circle in Figure 6D). As expected, LDH-Fe_3_O_4_-HA/DOX+HAase group exhibited the smallest tumor among all groups, and the tumor weight was about 4-fold lighter than that of LDH-Fe_3_O_4_-HA/DOX and 3-fold lighter than that of free DOX, demonstrating that LDH-Fe_3_O_4_-HA/DOX possess a higher therapeutic efficacy than free DOX with the aid of HAase pretreatment. Importantly, LDH-Fe_3_O_4_-HA/DOX group showed a significant lower tumor weight than pre-HA group by the virtue of their targeting property (p < 0.001), consistent with the *in vitro* experiment result. In consideration of the steady growth of body weight and high survival rate, LDH-Fe_3_O_4_-HA/DOX+HAase displayed excellent biocompatibility and satisfactory tumor suppression effect.

Moreover, H&E and TUNEL staining of tumors were conducted to evaluate the therapeutic effect of different treatments (Figure 6F). For mice treated with saline, LDH-Fe_3_O_4_-HA and HAase, cancer cells arranged compactly and no obvious sign of necrosis was found. In contrast, the morphology of tumor cells in free DOX, LDH-Fe_3_O_4_-HA/DOX and LDH-Fe_3_O_4_-HA/DOX+HAase groups changed dramatically and cell nuclei started to damage, indicating an effective inhibition of tumor. The TUNEL staining of tumors in different groups showed a similar trend of necrotic areas, and the cell apoptosis rates were calculated for quantitative analysis (Figure S10). Impressively, the cell apoptosis of LDH-Fe_3_O_4_-HA/DOX+HAase was as high as 91.13%, which was significantly higher than that of LDH-Fe_3_O_4_-HA/DOX (68.43%, p < 0.001), HA pretreated group (36.85%, p < 0.001), and free DOX group (80.24%, p < 0.05). These results further verified that therapeutic effect of LDH-Fe_3_O_4_-HA/DOX could be highly improved by HAase pre-treatment. In addition, except the cardiotoxicity of DOX group, no obvious pathological abnormality and lesion were observed in the H&E-stained histological images of major organs (heart, liver, spleen, lung, and kidney) after different treatments (Figure S11), indicating the good biosafety and lower side effect of LDH-Fe_3_O_4_-HA/DOX than free DOX.

## Conclusions

In summary, LDH-Fe_3_O_4_-HA/DOX was synthesized as a targeted theranostic nanoplatform for enhanced *T_1_*-weighted MR imaging and chemotherapy of cancer cells overexpressing CD44 receptors. The formed LDH-Fe_3_O_4_-HA possessed good colloidal stability and biocompatibility, 10-fold improved *r_1_* relaxivity in comparison with Fe_3_O_4_ NPs (4.38 mM^-1^ s^-1^ *vs* 0.42 mM^-1^ s^-1^), and specific targeting to cancer cells overexpressing CD44 receptors. After loading DOX with a high encapsulating efficiency, LDH-Fe_3_O_4_-HA/DOX exhibited a pH-responsive and HAase-accelerated release behavior and targeted tumor inhibition effect *in vitro*. For *in vivo* theranostic applications, with the pretreatment of tumor tissue with HAase to degrade the overexpressed HA in extra-cellular matrix, the designed nanoplatforms have a better tumor penetration for significantly enhanced MR imaging of tumors and tumor chemotherapy with low side effects. Therefore, this work constructed a novel theranostic nanoplatform LDH-Fe_3_O_4_-HA/DOX and provided a useful strategy to enhance their MR imaging effect and therapeutic efficiency by combining HAase pretreatment.

## Supplementary Material

Supplementary experimental details, figures and table.

## Figures and Tables

**Table 1:** Zeta potential and hydrodynamic diameter of LDH, Fe_3_O_4_, LDH-Fe_3_O_4_, LDH-Fe_3_O_4_-APS, LDH-Fe_3_O_4_-HA and LDH-Fe_3_O_4_-HA/DOX.

Samples	ξ-potential (mV)	Hydrodynamic size (nm)	Polydispersity Index (PDI)
LDH	36.60 ± 0.09	107.21 ± 1.01	0.28 ± 0.01
Fe_3_O_4_	-30.20 ± 2.90	10.22 ± 0.03	0.55 ± 0.13
LDH-Fe_3_O_4_	26.54 ± 0.91	124.24 ± 2.18	0.16 ± 0.02
LDH-Fe_3_O_4_-APS	28.17 ± 0.12	267.32 ± 47.61	0.30 ± 0.16
LDH-Fe_3_O_4_-HA	-13.30 ± 1.57	325.10 ± 63.54	0.34 ± 0.21
LDH-Fe_3_O_4_-HA/DOX	-9.10 ± 0.51	345.47 ± 36.90	0.40 ± 0.06
