# Supplementary material for: LDH-stabilized ultrasmall iron oxide nanoparticles as a platform for hyaluronidase-promoted MR imaging and chemotherapy of tumors
Source: Theranostics. 2020 Feb 3;10(6):2791–802. doi: 10.7150/thno.42906 (PMC7052882; doi:10.7150/thno.42906)
Supplement: Supplementary file 1 — Supplementary experimental details, figures and table. [file thnov10p2791s1.pdf]

Supporting Information

LDH-stabilized ultrasmall iron oxide nanoparticles as a platform for hyaluronidase-promoted MR imaging and chemotherapy of tumors

Ni Zhang,[#1] *Yue Wang,*[#2] *Changchang Zhang,*[1] *Yu Fan,*[1] *Du Li,*[1] *Xueyan Cao,* [1] *Jindong Xia,*[2] *Xiangyang Shi,*[1] *Rui Guo* [*1]

[1] State Key Laboratory for Modification of Chemical Fiber and Polymer Materials, International Joint Laboratory for Advanced Fiber and Low-dimension Materials, College of Chemistry, Chemical Engineering and Biotechnology, Donghua University, Shanghai 201600, People's Republic of China

[2] Department of Radiology, Shanghai Songjiang District Central Hospital, Shanghai 201600, People's Republic of China

---

‡ These authors contributed equally to this work.

* To whom correspondence should be addressed. Tel: +86 21 67792750; Fax: +86 21 67792306 804.

E-mail addresses: ruiguo@dhu.edu.cn (R. Guo).

Part of experimental details

Materials

Iron(III) chloride anhydrous ($FeCl_3$), trisodium citrate dihydrate ($NaCit_3 \cdot 2H_2O$), sodium acetate anhydrous (NaAc), magnesium nitrate hexahydrate ($Mg(NO_3)_2 \cdot 6H_2O$), aluminum nitrate nonahydrate ($Al(NO_3)_3 \cdot 9H_2O$), sodium hydroxide (NaOH) and other agents were from Sinopharm Chemical Reagent Ltd. (3-aminopropyl)triethoxysilane (APS) and hyaluronidase (HAase) were obtained from Sigma-Aldrich (St. Louis, MO). Hyaluronic acid (HA, M_w = 5830) was obtained from Zhenjiang Dong Yuan Biotechnology Corporation (Zhenjiang, China). Doxorubicin hydrochloride (DOX·HCl) was purchased from HuaFeng Co., Ltd. (Beijing, China). 1-(3-Dimethylaminopropyl)-3-ethylcarbodiimide hydrochloride (EDC·HCl), and *N*-hydroxysuccinimide (NHS) were from GL Biochem. (Shanghai, China). Mouse melanoma cell line B16 was from Institute of Biochemistry and Cell Biology (the Chinese Academy of Sciences, Shanghai, China). RPMI-1640 medium, fetal bovine serum (FBS), penicillin, and streptomycin were purchased from Hangzhou Jinuo Biomedical Technology (Hangzhou, China). Cell counting kit-8 (CCK-8) was supplied by 7Sea Pharmatech Co., Ltd. (Shanghai, China). The water used in all experiments was purified using a Milli-Q Plus 185 water purification system (Millipore, Bedford, MA) and exhibited a resistivity greater than 18.2 MΩ·cm.

Characterization

The morphology and structure of LDH, Fe_3O_4, LDH-Fe_3O_4 nanoparticles were examined using a JEOL 2010F analytical electron microscope (JEOL, Tokyo, Japan) at an operating voltage of 200 kV. X-ray diffraction (XRD) analysis was carried out using a D/max 2550 VB+/PC X-ray diffractometer (Rigaku Cop., Tokyo, Japan) with Cu Kα radiation (λ = 0.154056 nm) at 40 kV and 200 mA and a 2θ scan range of 5-90°. Fourier transform infrared (FT-IR) spectra were recorded on a Nicolet 6700 FT-

IR spectrophotometer (Thermo Electron Corporation, Madison, WI). Samples were mixed with milled KBr crystals and pressed to form 13-mm diameter disks before measurements. A TG 209 F1 (NETZSCH Instruments Co., Ltd., Bavaria, Germany) thermogravimetric analyzer was used for thermogravimetric analysis (TGA) of the samples under nitrogen atmosphere in a temperature range of 10-900°C. Zeta potential and dynamic light scattering (DLS) measurements were conducted using a Malvern Zetasizer Nano ZS model ZEN3600 (Worcestershire, UK) equipped with a standard 633 nm laser. UV-vis spectrophotometer was carried out using a Lambda 25 UV-vis spectrophotometer (Perkin Elmer, Boston, MA). Mg, Al and Fe concentrations of these NPs in solutions were analyzed using a Leeman Prodigy inductively coupled plasma-optical emission spectroscopy (ICP-OES, Hudson, NH). T_1 and T_2 relaxometry were performed using a 0.5 T NMI20 Analyzing and Imaging system (Shanghai NIUMAG Corporation, Shanghai, China). The parameters were set as follows: TR = 400 ms, TE = 20 ms, resolution = 156 mm × 156 mm, section thickness = 0.5 mm. The r relaxivity was obtained through linear fitting of the inverse T relaxation time ($1/T$) as a function of Fe concentration.

Drug loading

The DOX encapsulation efficiency and percentage can be calculated by measuring the concentration of free DOX in the collected supernatants after 3 times of centrifugation using a Lambda 25 UV-vis spectrophotometer (PerkinElmer, Waltham, MA) at 480 nm and a standard DOX absorbance-concentration calibration curve. The drug loading content (DL%) and entrapment efficiency (EE%) of nanodisks can be calculated using eqn (1) and (2), respectively.

$$DL\% = M_t / M_0 \times 100\% \quad (1)$$

$$EE\% = M_t / M_L \times 100\% \quad (2)$$

where M_t, M_0 and M_L stand for the masses of the encapsulated DOX, the initial DOX, and the drug loaded nanocomplexes, respectively.

***In vitro* drug release**

In vitro drug release profile of DOX from LDH-Fe_3O_4-HA/DOX was investigated in the presence or absence of HAase at pH 5.0 and 7.4 buffer solutions. LDH-Fe_3O_4-HA/DOX were dispersed in 1 mL corresponding buffer solution at the concentration of 1 mg/mL with/without HAase (1 mg/mL), and then sealed in a dialysis bag (cutoff Mw = 3500). The dialysis bag was immersed in 9 mL buffer solution (pH 5.0 or 7.4) and placed in shaker at 37°C for 48 h. Within a specified time interval (0.5, 1, 2, 4, 6, 9, 12, 24, 48 h), 1 mL sample solution was taken out from buffer solution to measure the released amount of DOX (λ_{DOX} = 480 nm) by ultraviolet-visible spectrophotometer, and an equivalent amount of fresh buffer solution was added.

LDH-Fe_3O_4-HA were dispersed in buffer solution of pH 5.0 and 7.4 (1 mg/mL), and then HAase (1 mg/mL) was added to degrade HA on the surface of NPs at 37°C under shaking. The zeta potential of LDH-Fe_3O_4-HA were measured by DLS at 15 min, 1 h, 2 h, 4 h, respectively.

***T_1* *and* *T_2* MR relaxometry and *T_1* imaging measurement.**

An NMI20-Analyst NMR Analyzing and Imaging system (0.5 T, Shanghai NIUMAG Corporation, Shanghai, China) was utilized to measure the T_1 and T_2 MR relaxometry of the Fe_3O_4, LDH-Fe_3O_4 and LDH-Fe_3O_4-HA NPs. First, these nanoparticles were formulated into the same iron concentration (0.1-1.6 mM), then the transverse relaxivity (r_1 and r_2) was calculated by linear fitting the inverse T_1 and T_2 relaxation time ($1/T_1$ and $1/T_2$) as a function of Fe concentration, respectively. Finally, according to their relaxation rate, comparing the introduction of LDH and HA has a positive effect on the relaxation rate of contrast agent. Consistent with the above results, concentration

dependent brightening effects were also observed by T_1-weighted MR images under the same gradient concentration conditions.

Cytotoxicity and cellular uptake assays

B16 melanoma cells with CD44 receptors over-expressed were cultured in 1640 medium supplemented with 10% FBS, 100 U/mL penicillin, and 100 U/mL streptomycin in a Thermo Scientific cell incubator at 37°C and 5% CO_2 (Waltham, MA). For pre-HA group, B16 cells were pre-treated with HA solution (2.0 mM) for 2 h before the addition of materials in order to block the CD44 receptors on cell surface.

The cytotoxicity of LDH-Fe_3O_4-HA was evaluated by CCK-8 assay. Typically, B16 cells were grown in 96-well plate with a density of 1×10^4 cells per well. When the cells were completely adhered, different concentrations of LDH-Fe_3O_4-HA (10, 20, 40, 80 and 100 μg/mL) medium solution were added into the plate (n = 6). After being incubated for 24 h, the medium was poured out and the cells were washed with PBS. Finally, the cells were incubated with 100 μL of serum free medium containing 10 μL of CCK-8 solution at 37°C for another 4 h in a dark environment. The absorption value at 450 nm was determined by a microplate reader (Multiskan MK3, Massachusetts, Thermo).

In order to evaluate the inhibition effect of LDH-Fe_3O_4-HA/DOX, B16 cells (1×10^4 cells/well) were cultured in 96-well plate overnight. Next, the cells were washed with PBS, and then the new medium containing different concentrations PBS, free DOX and LDH-Fe_3O_4-HA/DOX (c_{DOX} = 1.6, 3.2, 6.3, 12.5, 25 μg/mL) were added and cultured for 24 and 48 h. Finally, the absorbance values of different groups were determined by CCK-8 assay, and the cell survival rate was calculated.

The cellular uptake of LDH-Fe_3O_4-HA NPs by B16 *in vitro* was quantitatively evaluated by measuring the Fe concentration in cells using inductively coupled plasma-optical emission

spectroscopy (ICP-OES, Hudson, NH). B16 cells were seeded into 12-well plates at a density of 2 × 10^5 cells per well and cultured overnight to lead cells to adherence. Then, the medium was replaced with fresh medium containing LDH-Fe_3O_4-HA NPs at different Fe concentrations (5, 10, 20 and 50 μg/mL) and cells were further incubated for 4 h. Afterwards, the medium was removed carefully and cells were washed 3 times with PBS, treated with trypsin, suspended in fresh medium, and counted by hemocytometry. The remaining cells were collected by centrifugation (104 g, 5 min), and treated with 1.0 mL aqua regia solution for 24 h. Finally, the samples were diluted in PBS and measured the concentration of phagocytic segregants by ICP-OES.

Flow cytometry analysis and confocal microscopy

B16 cells were cultured in 12-well plate at a density of 2 × 10^5 cells per well. 1 mL LDH-Fe_3O_4-HA/DOX medium solution (c_{DOX} = 5, 10, 15, 20 and 25 μg/mL) was added to each well and incubated for 4 h. PBS medium solution was set as a blank control group. Then, carefully pouring out LDH-Fe_3O_4-HA/DOX medium solution, cells were separated, collected, and washed repeatedly with PBS by centrifugation. Finally, cells were suspended in PBS and analyzed using a Becton Dickinson FACScan flow cytometer (BD Biosciences, Franklin Lake, NJ).

For confocal laser scanning microscope (CLSM, Zeiss, Jena, LSM700) observation, B16 cells were seeded in a petri dish with 20 mm^2 glass bottom at a density of 1 × 10^5 per dish and incubated for 24 h. Subsequently, B16 cells were co-cultured with free DOX, LDH-Fe_3O_4-HA/DOX (c_{DOX} = 5 μg/mL) for 4 h, respectively. The cells were lightly washed with PBS buffer solution, fixed with paraformaldehyde for 15 min and stained with DAPI for 30 min. Finally, CLSM was used to directly observe intracellular uptake and intracellular distribution of free DOX and drug-loaded nanocarrier.

***In vivo* MR imaging and biodistribution**

All animal experiments were conducted under the direction of the Ethical Committee of Shanghai Songjiang District Central Hospital, and according to the policy of the National Ministry of Health. To set-up tumor xenograft model, about 2×10^6 cells suspended in saline (100 μL) were subcutaneous injected in the right leg of C57BL/6 mice (20-25 g, 6 weeks, Shanghai Slac Laboratory Animal Center, Shanghai, China). When the volume of the tumor reached 200 mm^3, mice were randomly divided into 4 groups. B16 tumor-bearing mice were anesthetized by intraperitoneal injection of pentobarbital sodium (40 mg/kg for each mouse), and then were intravenously injected with LDH-Fe_3O_4-HA NPs ([Fe] = 500 μg/mL, 0.2 mL in saline for each mouse). For pre-HA and HAase group, HA (24 mg, 100 μL saline) and HAase (0.1 mg, 50 μL saline) were intratumorally injected 1 h before the intravenous injection of LDH-Fe_3O_4-HA, respectively. To evaluate the targeted property of LDH-Fe_3O_4-HA after HAase pretreatment, free HA was injected 1h after the administration of HAase at tumor, and then LDH-Fe_3O_4-HA nanoparticles were injected intravenously. T_1-weighted MR images were obtained after intravenous injection at the time points of 0, 5, 15, 30 and 60 min, respectively. Eventually, MR scanning of the mice was carried out using a 3.0 T Signa HDxt superconductor MR system (GE Medical Systems, Milwaukee, WI). The T_1- weighted MR images were recorded with the following parameters: TR = 1959 ms; TE = 16.1 ms; point resolution = 256 mm × 256 mm; section thickness = 1 mm; FOV = 80 × 80 mm; and number of excitations = 1.

After the mice were sacrificed at the given time points (15 min, 60 min and 24 h, respectively), the heart, liver, spleen, lung, kidney and tumor were extracted. These organ and tumor samples were weighed and digested by aqua regia solution for a week. After complete tissue digestion, each sample was diluted by water to 4 mL. Then, the Fe content in these samples was measured by ICP-OES for 3 times and the data were expressed as mean ± SD (n = 3).

In vivo antitumor activity

B16 tumor-bearing mice were divided into 7 groups randomly and intratumorally administrated with saline, LDH-Fe_3O_4-HA, free DOX (5 mg/kg), LDH-Fe_3O_4-HA/DOX+pre-HA (5 mg/kg), LDH-Fe_3O_4-HA/DOX (5 mg/kg), HAase, LDH-Fe_3O_4-HA/DOX+HAase (5 mg/kg) on day 1, 4, and 7. For pre-HA and HAase group, HA (24 mg, 100 μL saline) and HAase (0.1 mg, 50 μL saline) were intratumorally injected 1 h before the administration of NPs, respectively. The tumor volumes and the body weights were recorded every two days. The tumor volume was calculated according to a formula of $V = W^2 \times L/2$, where W and L represent the width and length of tumor. The relative tumor volume was calculated based on the tumor volume of the first day. After 10 days, tumors and major organs were excised from euthanized mice, photographed and weighed, followed by hematoxylin and eosin (H&E) staining. Standard TdT mediated dUTP nick-end labeling (TUNEL) staining tests were performed according to our previous work to confirm the tumor cell apoptosis efficacy.

Statistical analysis

One-way ANOVA statistical analysis was used to analyze the significance of the experimental data. A p value of 0.05 was selected as the level of significance, and the data were indicated with (*) for $p < 0.05$, (**) for $p < 0.01$, and (***) for $p < 0.001$, respectively.

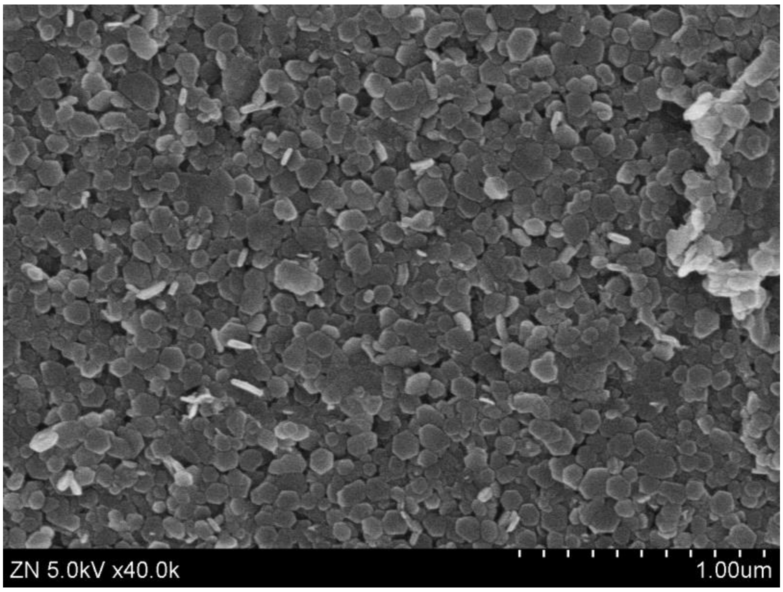

Figure S1. SEM image of LDH NPs.

Table S1. The 2*θ* peak position and plane spacing XRD diffraction patterns results of LDH and $LDH-Fe_3O_4$ NPs.

**diffraction plane (*hkl*)**	**2*θ* peak position (deg)**		**plane spacing (*d*, Å)**	
	LDH	$LDH-Fe_3O_4$	LDH	$LDH-Fe_3O_4$
(003)	11.30	11.44	7.82	7.73
(006)	23.14	22.78	3.84	3.90
(012)	34.46	34.76	2.60	2.58
(015)	38.88	38.54	2.31	2.33
(018)	46.64	46.03	1.95	1.97
(110)	60.70	60.72	1.52	1.52

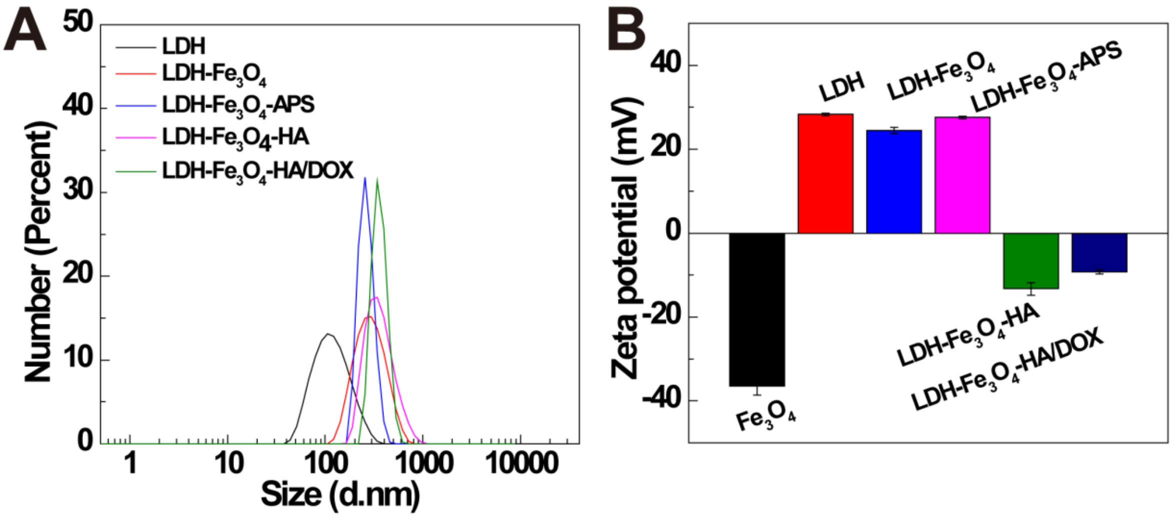

Figure S2. (A) Hydrodynamic diameters and (B) zeta potentials of LDH, Fe_3O_4, LDH-Fe_3O_4, LDH-Fe_3O_4-APS, LDH-Fe_3O_4-HA and LDH-Fe_3O_4-HA/DOX NPs.

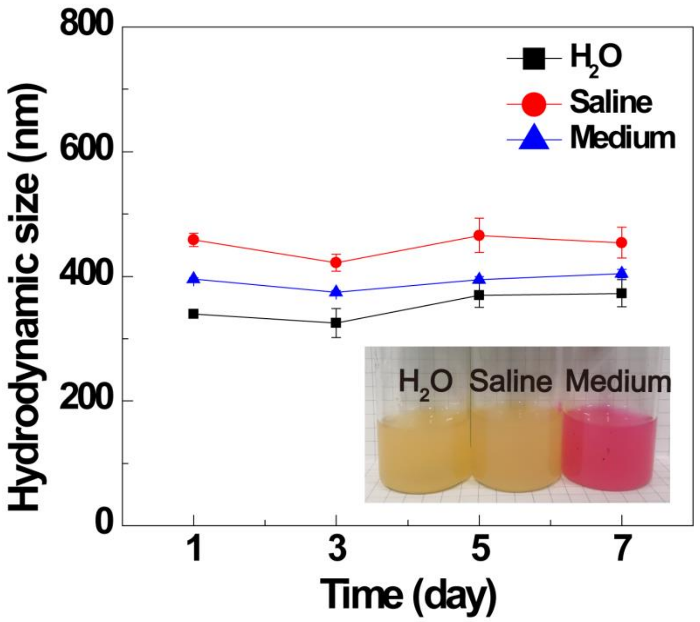

Figure S3. The changes of hydrodynamic diameters of LDH-Fe_3O_4-HA dispersed in water, saline, and RPMI 1640 medium (containing 10% FBS) for 1, 3, 5 and 7 days. Photograph of LDH-Fe_3O_4-HA dispersed in these solutions for one month.

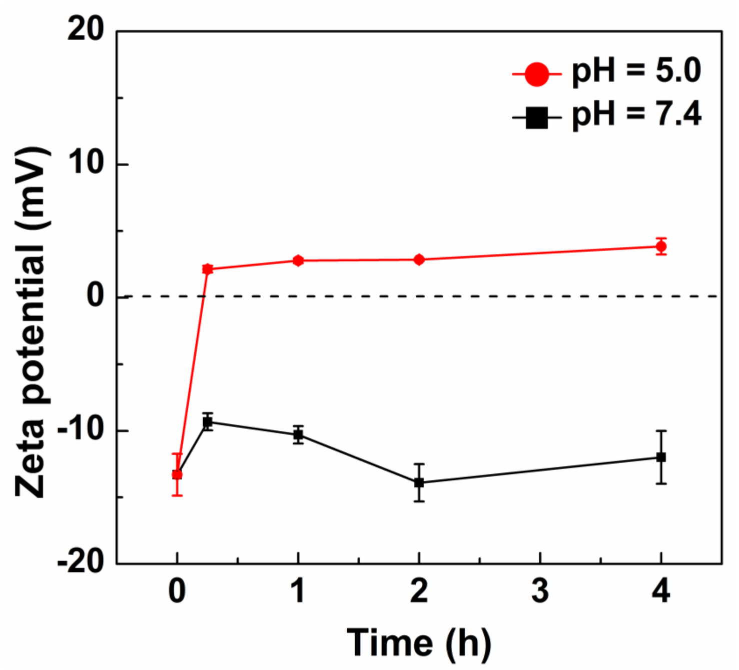

Figure S4. The zeta potential of LDH-Fe_3O_4-HA after incubated with HAase (1 mg/mL) for different time at pH 5.0 and 7.4, respectively.

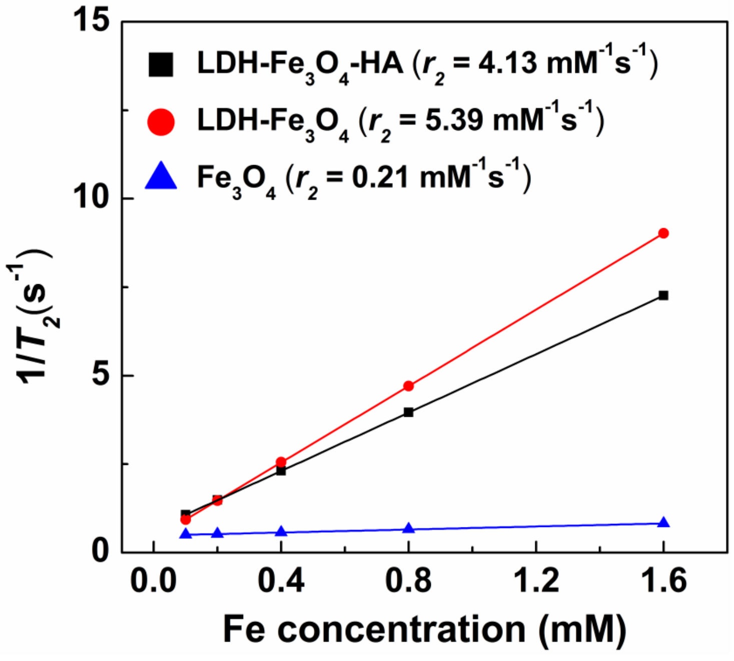

Figure S5. The linear fitting of $1/T_2$ in terms of Fe concentration of Fe_3O_4, LDH-Fe_3O_4 and LDH-Fe_3O_4-HA solutions at different Fe concentration (0.1, 0.2, 0.4, 0.8, and 1.6 mM).

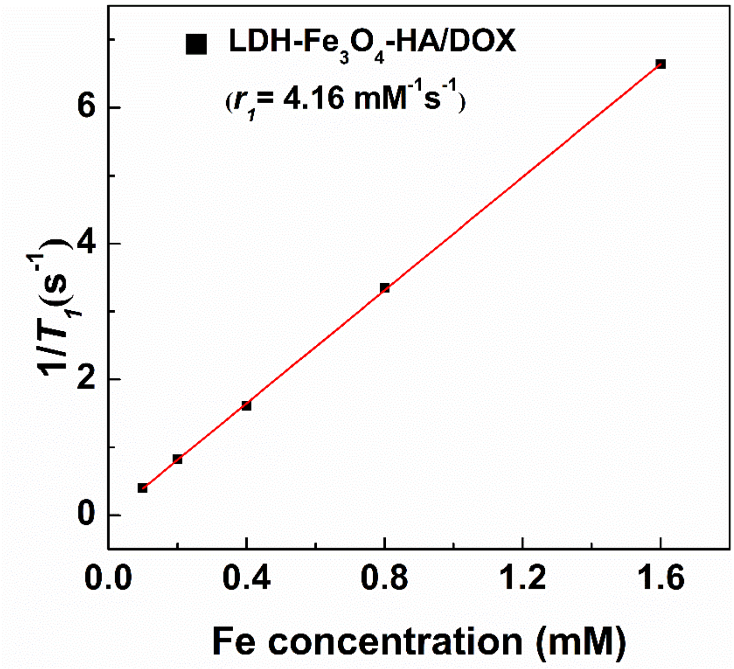

Figure S6. The linear fitting of $1/T_1$ in terms of Fe concentration of LDH-Fe_3O_4-HA/DOX solutions at different Fe concentration (0.1, 0.2, 0.4, 0.8, and 1.6 mM).

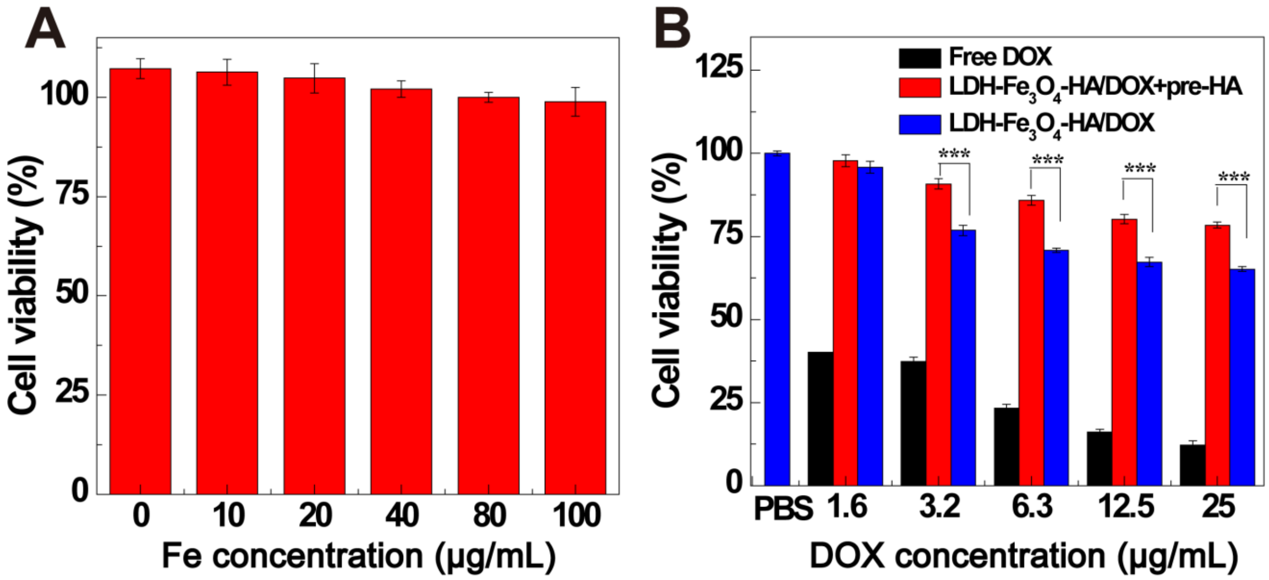

Figure S7. (A) CCK-8 assay of B16 cells after incubation with different concentrations of LDH-Fe_3O_4-HA for 24 h; (B) viability of B16 cells treated with free DOX and LDH-Fe_3O_4-HA/DOX at different concentrations of DOX for 24 h.

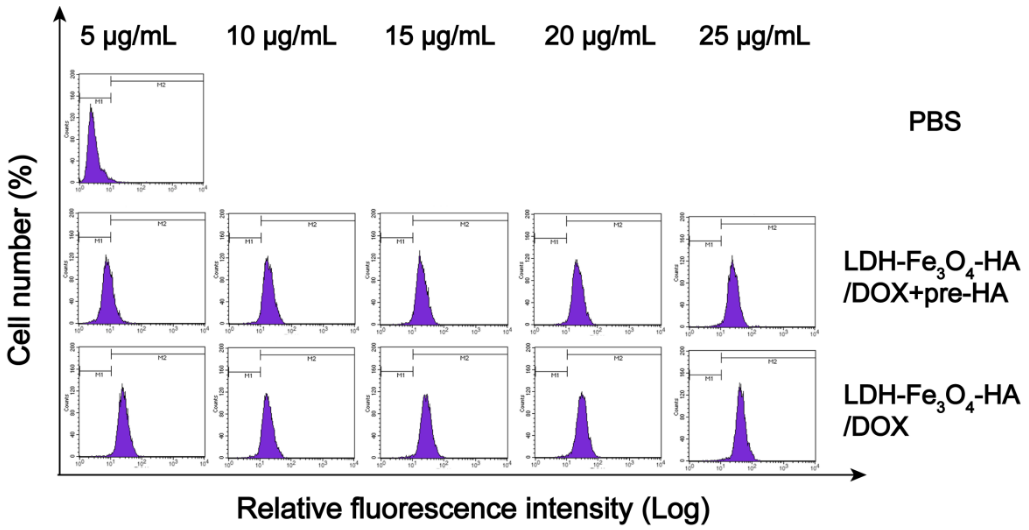

Figure S8. Flow cytometry results of PBS and LDH-Fe_3O_4-HA/DOX in presence and absence of HA in the medium.

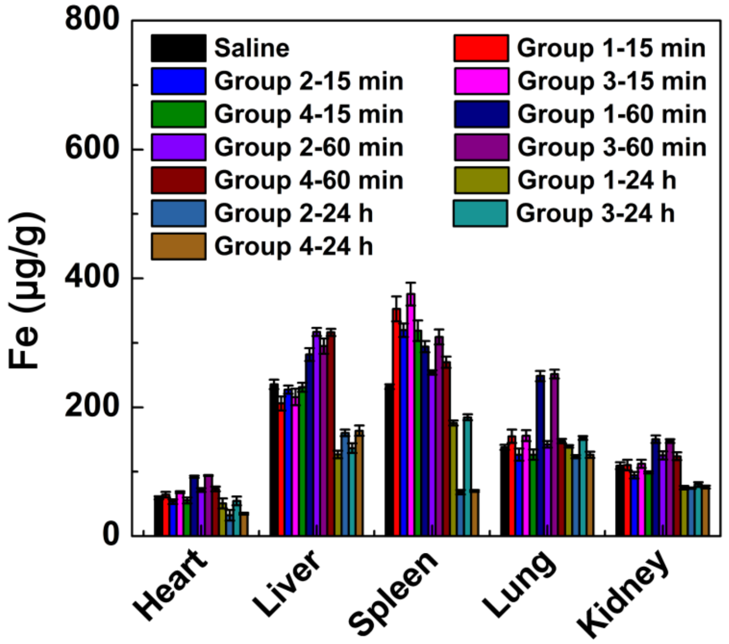

Figure S9. Biodistribution of Fe in the major organs after administration (Group 1: LDH-Fe_3O_4-HA+HAase; Group 2: LDH-Fe_3O_4-HA+HAase+pre-HA; Group 3: LDH-Fe_3O_4-HA; Group 4: LDH-Fe_3O_4-HA+pre-HA).

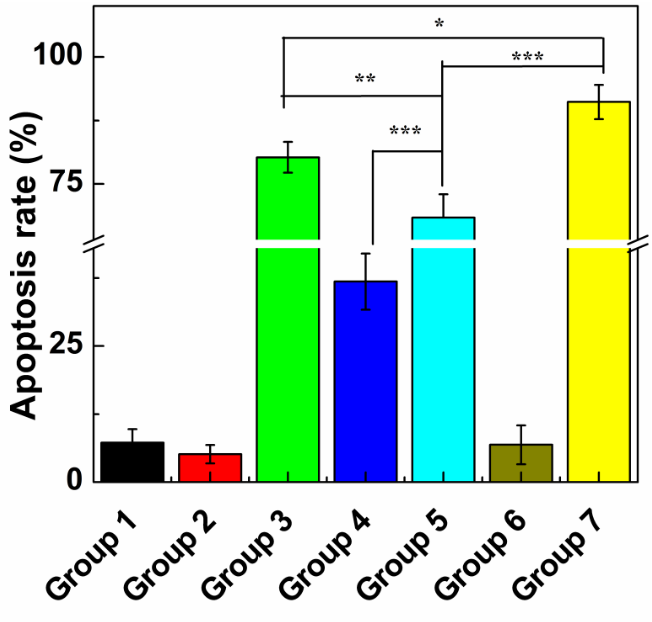

Figure S10. Apoptosis rate of tumor after different treatments. Group 1, saline; Group 2, LDH-Fe_3O_4-HA; Group 3, free DOX; Group 4, LDH-Fe_3O_4-HA/DOX+pre-HA; Group 5, LDH-Fe_3O_4-HA/DOX; Group 6, HAase; Group 7, LDH-Fe_3O_4-HA/DOX+ HAase.

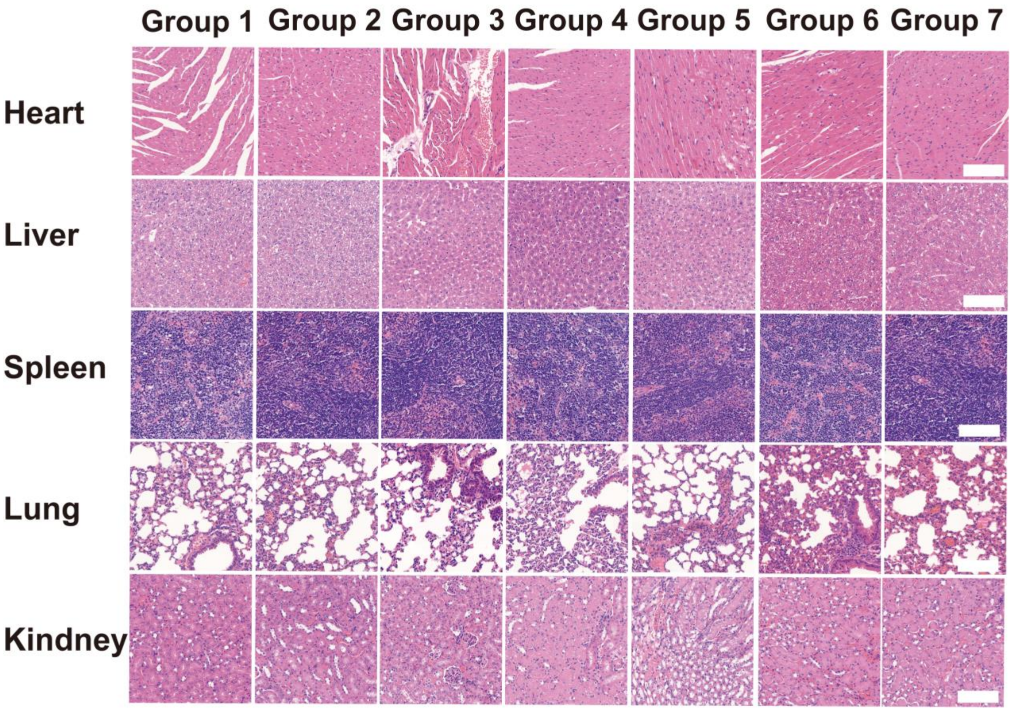

Figure S11. H&E-stained histological images of major organs after different treatments (scale bar: 100 μm).
